# Challenges to rutile-based geoscientific tools: low-temperature polymorphic TiO_2_ transformations and corresponding reactive pathways

**DOI:** 10.1038/s41598-020-64392-8

**Published:** 2020-05-04

**Authors:** André Jorge Pinto, Nuria Sanchez-Pastor, Ivan Callegari, Bernhard Pracejus, Andreas Scharf

**Affiliations:** 1 Applied Geology Department, German University of Technology in Oman, P.O. Box 1816, PC 130 Athaiba, Sultanate of Oman; 2 Department of Mineralogy and Petrology, Faculty of Geological Sciences, University Complutense of Madrid, C/ José Antonio Novais, 12, Madrid, 8040 Spain; 3 Department of Earth Sciences, Sultan Qaboos University, Al-Khoud, Oman

**Keywords:** Geochemistry, Mineralogy

## Abstract

Rutile, a common accessory mineral in a wide variety of rocks, is the most stable naturally occurring TiO_2_ polymorph. The relationship between its trace element composition and formation conditions has provided geoscientists with discriminant tools for fingerprinting geological processes, such as magmatic evolution and subduction zone metamorphism, alongside applications to the study of sediment provenance. In the present work, volcaniclastic rock samples belonging to Fara and Saiq Formations, outcropping in Jebel Akhdar mountains, Oman, are studied with Raman spectroscopy and Electron Microprobe (EMP) aiming: of (i) the identification of different naturally-occurring TiO_2_ polymorphs, (ii) the evaluation of their trace element contents in relation with hydrothermal alteration features, and (iii) the analysis of the mineral reactive pathways behind the observed textural relationships. Raman investigations demonstrated that interstitial, fine-grained TiO_2_ corresponds to anatase, whereas rutile occurs as isolated single grains. EMP determinations further revealed that an identified Nb-enrichment in anatase is coupled with a corresponding Nb-depletion in rutile. The combination of the obtained results with petrographic observations enabled unravelling the TiO_2_ reactive pathways affecting the studied samples. Thus, a coupled polymorphic dissolution-precipitation reaction assisting rutile-to-anatase conversion has been defined, together with the role of Nb in further stabilizing the structure of the lower temperature polymorph. Semi-quantitative thermometric considerations suggest that rutile substrates are likely of magmatic origin, whereas anatase formation is clearly associated with a lower temperature aqueous environment. The gathered results raise fundamental questions concerning the application of commonly used rutile-based geochemical and thermometric tools.

## Introduction

The natural abundance of TiO_2_ in geologic materials is dominated by the most stable rutile polymorph, which occurs in a wide range of rock types (igneous, metamorphic, and sedimentary) as an accessory mineral. The chemical variability of naturally occurring rutile has been recognized since long ago^1–5^, especially concerning the replacement of Ti by high field strength elements (HFSE), such as Nb and Ta, of which it is a major host phase. Thus, the trace element contents of rutile provide a useful tool for geochemically fingerprinting processes such as magmatic evolution or high-pressure metamorphic systems^6,7^. A remarkable application of rutile trace element chemistry is the use of Nb and Cr contents for discriminating different rutile source lithologies in provenance analysis investigations^8–10^, coupled with Zr-in-rutile thermometric determinations^11–13^. Expanding on previous applications, Pape and co-workers^14^ assessed the Zr-in-rutile temperature-dependent relationships in ultra-high pressure (UHP) conditions.

The study of hydrothermal rutile knows fewer efforts in comparison to both metamorphic and magmatic origins, as indicated by scarcer references^15–19^. These studies focused on the comparative evaluation of different thermometric techniques applied to the crystallization of rutile in hydrothermal quartz-dominated veins. For instance, Cabral *et al*.^18^ compared the crystallization temperatures of hydrothermal rutile obtained independently through fluid inclusion analysis and the application of Zack et al.^11^ Zr-in-rutile geothermometer, revealing overestimated deviations of the latter under the studied low-temperature ranges. Finally, an extensive compilation and discussion of rutile applications in Earth Sciences is included in Meinhold et al.^20^.

Rutile is also an economically relevant industrial raw material, having attracted research efforts not only due its employment in the production of white pigments^21^, but also applications as a photocatalyst, photoelectrode, gas sensor and bioactive material^22–25^. The main economical source of this mineral is globally exploited as rutile-enriched sands^26,27^.

Anatase has not received the same degree of attention from geoscientists, most likely because of its association with low-temperature, aqueous superficial environments^28–30^. However, the physical-chemical properties of anatase doped with different trace elements have been addressed by a large number of investigations^31–33^, regarding engineering solutions in the energy and environmental sectors, ranging from photocatalysis^34^ to lithium battery anodes^35^. Likewise, the temperature-induced phase transition of anatase to rutile has been researched under different experimental conditions^36–38^, but studies concerning rutile to anatase conversion are strikingly absent from the scientific literature. Additionally, the impact of such mineral replacement reactions over the application of commonly used rutile-based geoscientific tools remains unknown.

In the present work, we focus on the mineralogical and chemical characterization of TiO_2_ phases included in volcaniclastic rocks belonging to Fara and Saiq formations (Jebel Akhdar mountains, Oman) with the purpose of: i) identify the different naturally-occurring TiO_2_ polymorphs, ii) evaluate their trace element contents in relation with hydrothermal alteration features, and iii) decipher the mineral reactive pathways behind the observed textural relationships.

### Geological setting

**Figure 1:**
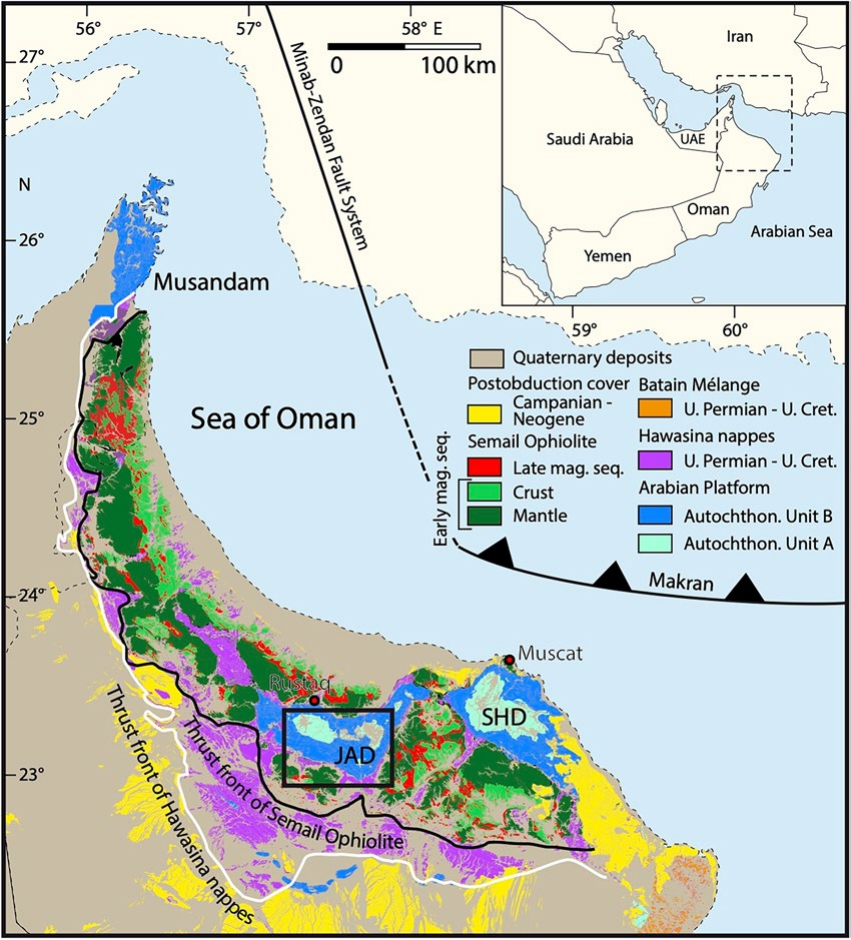
Tectonic overview map of the northeastern Arabian Peninsula. JAD – Jabal Akhdar Dome; SHD – Saih Hatat Dome. Map modified after Béchennec et al. (1993) and Callegari et al. (2020). The black dashed rectangle in JAD depicts the area of sampling (Fig. 2a–c). Mag. Seq. = magmatic sequence.

**Figure 2:**
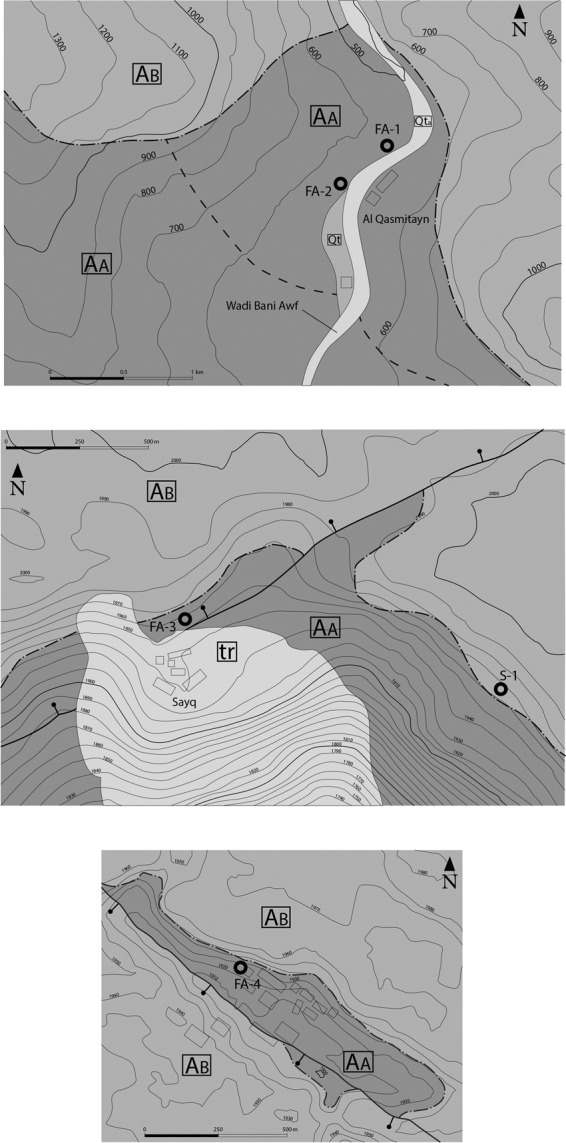
Geological maps and sample collection sites below and above the unconformity (dash and dot black line) at (**a**) Wadi Bani Awf and (**b,c**) Saiq Plateau locations. AA: Autochthonous A, AB: Autochthonous B, Qt: Quaternary inactive alluvial deposits, Qta: Quaternary active deposits, tr: travertine deposits. In Fig. 2a: the dashed black line marked the boundary between Neoproterozoic (to the SW) and Cambrian rocks in Fara Formation. All representations of geological elements resulted for the present´s work field studies.

The Oman Mountains comprise a thick Neoproterozoic to Neogene siliciclastic and carbonate rocks succession divided in two tectonic units. The Neoproterozoic to Carboniferous rocks (known as the “Autochthonous Unit A”^39^) are only exposed at the cores of the Jabal Akhdar (JAD) and Saih Hatat (SHD) domes (Fig. 1). The Autochthonous Unit B represents the Permo-Mesozoic Arabian shelf sedimentary rocks (Hajar Supergroup). The Autochthonous units A and B are separated by an angular unconformity, the so-called “Hercynian” Unconformity (Fig. 2) whose geological significance is still a matter of debate. This unconformity is considered an expression of the “Hercynian Orogeny^40^, related to the collision between Gondwana and Laurasia supercontinents. Nevertheless, this “Hercynian” event has been recently interpreted to be mainly a thermal one^41^ without significant deformation. Thus, the absence of a “Hercynian” deformation record leaves the age of the unconformity unsettled^42^. The formations belonging to Autochthonous Units A and B in the JAD (Fig. 1) nearby the “Hercynian” Unconformity were the sampling target of the present study (Fig. 1). These comprise volcaniclastic rocks belonging to the Upper Member of Fara (beneath the unconformity) and Saiq (above the unconformity) Formations, outcropping in the western and eastern sectors, respectively, of the Jebel Akhdar Mountains in Oman.

The Fara Formation outcrops in Wadi Bani Awf (WBA type locality, Fig. 2), in the western sector of JAD and is unconformably overlain by Saiq Formation. It has been divided into three members^40^: the lower 60 m-thick member consists of black chert and ignimbrites; the 200 m-thick middle member contains conglomerate, siltstone, breccia, sandstone, sandy limestone, stromatolites and abundant phosphate grains; and the 120 m-thick upper member comprises quartzose siltstone, gray-green argillaceous siltstone, green silicic tuffite, graywacke, sandstone and carbonate-cemented conglomerate^39^. The Fara Formation volcaniclastic rocks in WBA were the subject of past geochronological studies yielding 544.5 ± 3.3 Ma U-Pb zircon ages^43^.

In the JAD, the Saiq Formation consists of two members. The lower member is represented by a < 25 m-thick sequence of conglomerate, sandy limestone, siltstone, felsic volcaniclastic and volcanic rocks, whereas the upper ~400 m-thick member is composed mostly of fossil-rich lithologies (e.g., corals, foraminera, gastropods, echinoderms), shallow-marine dark limestone and dolostone towards the top^44^.

Four samples of volcaniclastic rocks were collected below the “Hercynian” Unconformity at Wadi Bani Awf (FA-1 and 2, Fig. 2a) and Saiq Plateau locations (SP) (FA-3 and 4, Fig. 2b and c). For contrasting purposes, the volcaniclastic materials immediately overlying the unconformity were also sampled at SP (S-1, Fig. 2b).

## Experimental

Fresh samples collected at Wadi Bani Awf (WBA) and Saiq Plateau locations (SP) were embedded in resin, polished and studied with a petrographic microscope, under both transmitted and reflected light observation modes.

The Raman spectra of targeted phases were collected using a confocal Thermo Fischer DXR Raman Microscope, enabling a point-and-shoot Raman capability of one-micron spatial resolution. The selected objective was of x10 magnification coupled with a laser source 532 nm at 10 mW in a laser mode power of 100%. The average spectral resolution of the Raman shift ranging from 70 to 3400 cm^−1^ was 2–4 cm^−1^, i.e. grating 900 lines/mm and a spot size of 1–2 μm. The system was operated with OMNIC 1.0 software fitting working analyzing conditions, such as pinhole aperture of 25 µm and bleaching time of 1–2 s; four exposures averaged in time of 30 s each. Band component analysis was performed with software package “Fityk”^45^, which enables assessing Raman spectroscopic data by the employment of different functions. Furthermore, the software allows fixing or varying fitting function parameters such as height, center and hwhm (half width at half maximum) of the bands.

After marking target zones for analysis, the polished thin-sections were carbon coated prior to Electron Microprobe (EMP) determinations. The employed EMP was a JEOL Superprobe JXA-8900M equipped with five WDS spectrometers, an EDS spectrometer, and a backscattered electron detector (BSE), enabling a fine selection of the surfaces to be analyzed. Standard analyses applied 5 μm of beam diameter at a beam time current of 10 nA at 20 kV. Counting times on peaks and background ranged from 15 to 60 s and 5 to 30 s, respectively. The calculated detection limits were 56 ppm for Si (albite standard), 104 ppm for Fe (almadine standard), 66 ppm for Ti (titanium metal standard), 160 ppm for Zr (zirconium dioxide standard), 47 ppm for Al (sillimanite standards), 170 ppm for Ta (lithium tantalate standard), 68 ppm for Cr (chromium metal standard) and 102 ppm for Nb (lithium niobate).

## Results

### Sample Petrography

**Figure 3:**
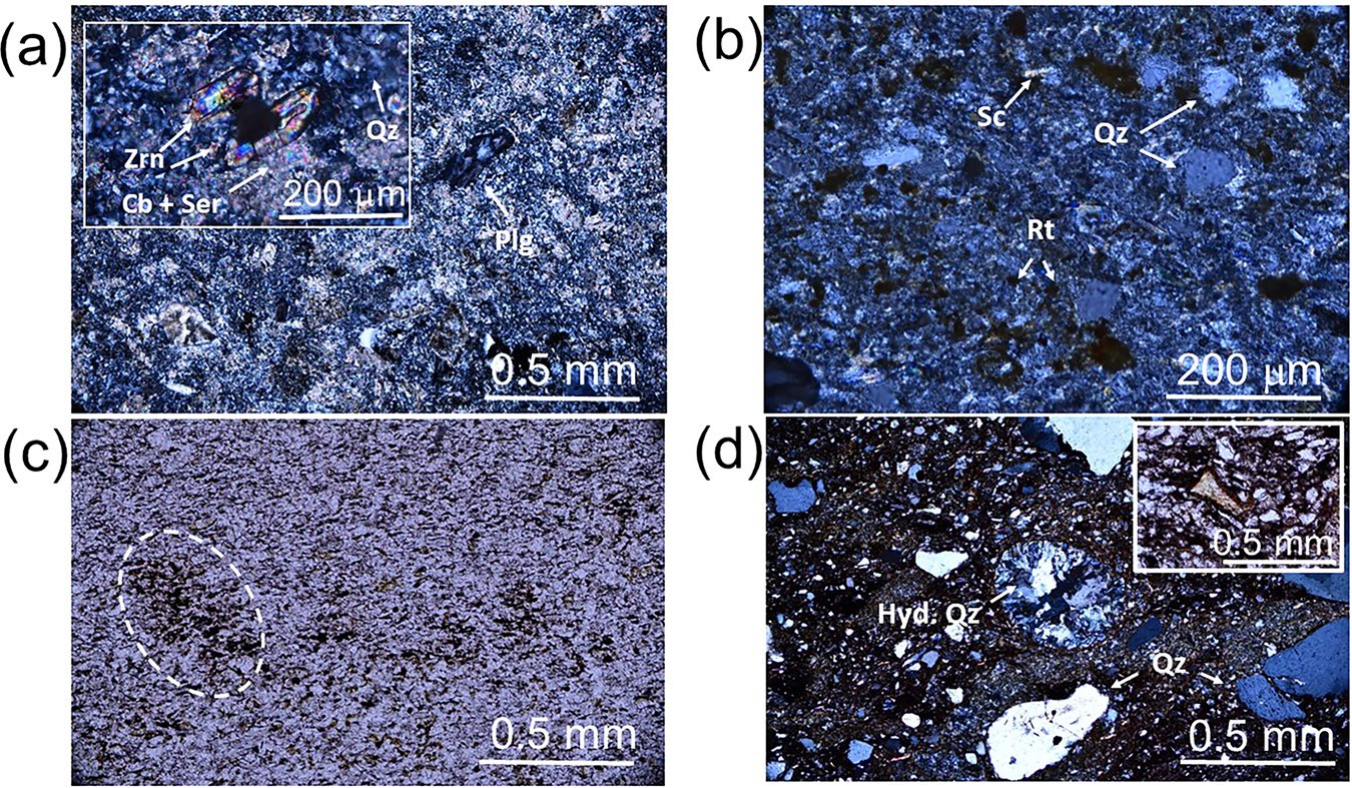
Optical micrographs of volcaniclastic rocks from the Upper Member of Fara Formation: (**a,b**) zircon-bearing ash tuffs (crossed polarizers, Wadi Bani Awf), (**c**) carbonatized lithic ash (?) (plane-polarized light, Saiq Plateau), (**d**) welded ash tuff (crossed polarizers, Saiq Plateau). The dashed ellipsis encloses a TiO_2_-rich area. Qz=Quartz, Hyd. Quartz= Hydrothermal Quartz, Zrn=Zircon, Cb=Carbonates, Ser=Sericite, Plg=Plagioclase, Rt=Rutile.

Figure 3 depicts optical micrographs of the sampled volcaniclastic rocks belonging to the Upper Member of Fara Formation, outcropping at Wadi Bani Awf and Saiq plateau. The fine-grained rocks from WBA (Fig. 3a,b) display abundant sub-angular to sub-rounded quartz clasts, at times displaying embayments, devitrified glass fragments and relicts of plagioclase/alkaline feldspar. Zircon grains, in most instances displaying elongated sections with curved edges, transversal cracks and diameters around 200 μm, are abundant in these lithologies (inset of Fig. 3a), and were the subject of past geochronological studies^43,46^. The overall primary mineralogical and textural arrangements are affected by widespread sericitization, silicification, carbonation, as depicted in Fig. 3a,b and the related hydrothermal alteration products correspond to the main matrix components. Furthermore, fine-grained saussuritization assemblages (quartz+carbonate+epidote) were found within relicts of glass fragments, where hydrothermal mineral replacement is more extensive, and occasionally affecting plagioclase relicts. The opaque minerals present in the studied samples from WBA include goethite, single-grained rounded to sub-rounded TiO_2_ phases (Fig. 3b) and fine-grained, interstitial TiO_2_ phases. Given the observed overall detrital grain size (<<2 mm), these rocks should correspond to ash tuffs. The larger grain-sized rutile, with clear tetragonal outlines, contains large pores and/or holed cores filled with quartz and/or carbonates. An incipient orientation of prismatic/tabular textural elements along with indented contacts between clasts, suggest a slight deformation print on these rocks. Finally, no autoclasts or other lithoclasts were found in these rocks, pointing towards a low degree of sedimentary reworking, and the two sampled rock-types revealed only variations in granularity, while sharing similar mineral contents.

Figure 3c,d display optical micrographs obtained for rock samples cropping out at Saiq Plateau locations, below the “Hercynian” Unconformity. Figure 3c refers to a fine-grained, highly carbonatized lithology, rich in opaque minerals, such as manganese oxides and hematite, but scarce TiO_2_ phases. The overall grain size involves diameters ≤ 10 μm, and there is a noticeable oriented fabric of prismatic shaped-carbonates, which suggests these may correspond to relicts of plagioclase grains. The granular elements of this rock are well calibrated, and though an accurate classification is difficult because of the intense hydrothermal alteration, the grain-size, monotonous mineral composition, and observed small-scale foliation seem to point towards lithified ash as the original lithology. The poorly sorted volcaniclastic rock of Fig. 3d reveals sub-angular to rounded, submillimeter-sized clasts of quartz frequently embayed, alkaline feldspar and lithoclasts containing fine lath-like relics of sericitized plagioclase. The abundant relics of glass shards have been devitrified to mixtures of chlorite, actinolite-tremolite amphibole and hematite, as shown on the inset in Fig. 3d, and are “plastically” accommodated to the outlines of detrital components in the rock. An amygdale filled with hydrothermal quartz is displayed in the center area of Fig. 3d, a further evidence of the volcanic nature of this lithology. The present opaque phases correspond to very fine-grained specularitic hematite and rare, fine-grained TiO_2_ phases (5 < d < 10 μm). Finally, the ferruginous cement materializes sub-parallel planar features suggesting an incipient foliation development, deformed around the clastic components, as seen at the lower boundary of the aforementioned amygdale. The general submillimeter size of the detrital fraction is compatible with an ash tuff classification. Owing to their stratigraphic position (Neoproterozoic and/or lowermost Cambrian) and lithological affinities with Upper Member Fara Formation volcaniclastics in WBA, these lithologies have been considered as Fara Formation equivalents in SP locations.

**Figure 4:**
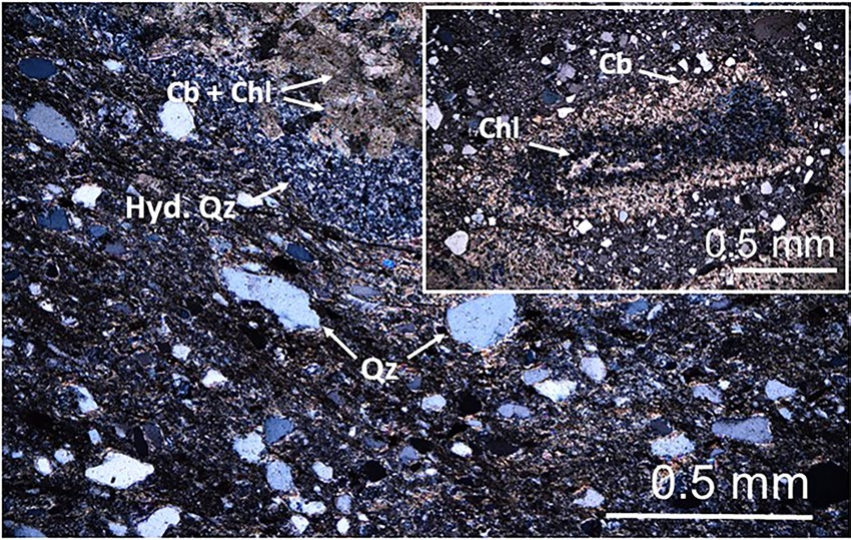
Optical micrograph under cross-polarized light of reworked welded ash-tuff belonging to the basal section of Saiq Formation (Saiq Plateau). Qz=Quartz, Hyd. Quartz= Hydrothermal Quartz, Cb= Carbonates, Chl=Chlorite.

An optical micrograph under crossed-polarized transmitted light of the volcaniclastic lithology found at the base of the post-unconformity Saiq Formation may be observed in Fig. 4. The detrital fraction comprises submillimeter-sized, rounded to sub-angular quartz, and highly chloritized/carbonatized/silicified lithoclasts, including autoclasts and relics of glass shards, with diameters up to ~2 mm, embedded in a matrix of fine-grained quartz and carbonates. In many instances, there is a mineralogical zonation of the replacement products affecting lithoclasts and relics of glass fragments. The inset of Fig. 4 displays a glass shard relic with a chlorite-rich core bounded by a carbonate-rich rim, and axiolitic microstructures associated to chloritization can also be found in other clasts. The opaque mineralogical content of this lithology consists of hematite, partially hematitized fine-grained pyrite, and coarse-grained, sub-rounded to sub-angular TiO_2_. Accounting for the prevalence of millimeter-sized lithoclasts and glass fragment relics (> 25%) this lithology should roughly correspond to a lapilli-ash tuff. In genetic terms, considering its stratigraphic position above the Neoproterozoic/lower Cambrian-Permian unconformity, this lithology displays mineralogical and textural affinities with the previously described tuffs from Fara Formation. Therefore, bearing in mind such an unconformity materializes an erosive surface, it is reasonable to assume that this rock includes materials eroded and re-worked from the underlying Fara Formation volcaniclastic lithologies.

A brief summary of the sampled lithologies can be consulted in Table 1, alongside the main aspects of hydrothermal alteration.

**Table 1:** Summary of sampled lithologies. The information in italics concerns geological features above the “Hercynian” Unconformity. Cbt=Carbonation; Chlt=Chloritization; Sauss=Saussiritization; Serc=Sericitization; Sil=Silicification.

Reference	Lithology	Hydrothermal Alteration	Formation
*S-1*	*Lapilli-ash Tuff*	*Cbt+Chlt+Sil*	*Saiq Fm*
FA-4	Ash Tuff	Chlt+Serc+Sil	Fara Fm
FA-3	Lithified Ash (?)	Cbt	
FA-2	Ash tuff	Serc+Sil+Cbt +Sauss	
FA-1	Ash tuff	Serc+Sil+Cbt +Sauss	

### Raman spectroscopy

**Figure 5:**
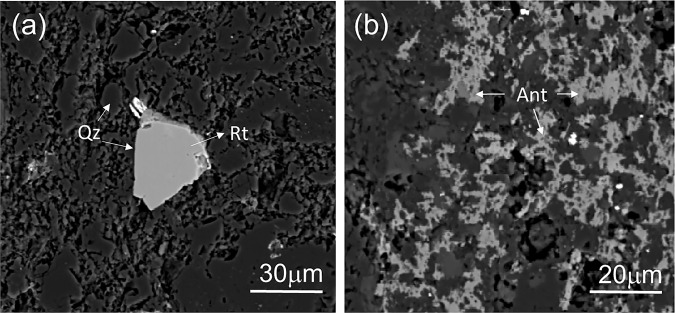
Back-scattered electrons micrographs of the different modes of TiO_2_ phases occurrence in the studied samples: (**a**) sub-angular single grains, and (**b**) interstitial fine-grained aggregates. Rt=Rutile; Ant=Anatase; Qz=Quartz.

Figure 5 displays the two modes of occurrence of TiO_2_ grains found in the studied samples. The coarser volcaniclastic rocks from Fara Formation (samples FA-1, FA-2 and FA-4) contain TiO_2_ phases occurring as single, sub-rounded to sub-angular grains with longer diameters inferior to ~10 μm (rarely, up to 25 μm), or fine-grained interstitial aggregates of grains concentrated throughout restricted volumes of the rocks. Finer-grained rocks belonging to Fara Formation cropping out in the Saiq sector (sample FA-3), comprise extremely low amounts of TiO_2_ minerals, as sub-rounded single grains interstitially dispersed in the overall mineralogical content. The lapilli-ash tuff from Saiq Formation (sample S-1) displays TiO_2_ phases as sub-angular to sub-rounded single grains, with maximum diameters of ~30μm. Nevertheless, no significant spectral differences were observed between these last and those belonging to Fara Formation, whereas different Raman signatures are clearly associated to TiO_2_ modes of occurrence as either single, isolated crystal grains or as fine-grained interstitial aggregates.

**Figure 6:**
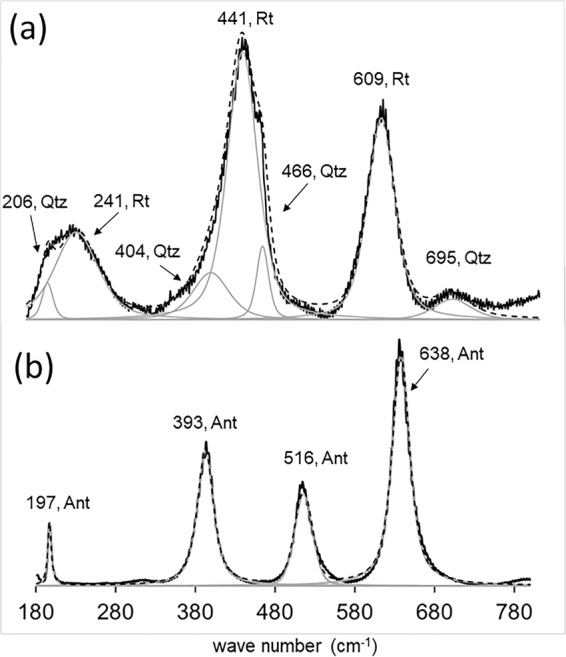
Representative Raman spectra of (**a**) a single TiO_2_ sub-rounded grain, and (**b**) fine-grained aggregate of interstitial TiO_2_ phases. Rt=Rutile; Ant=Anatase; Qz= Quartz.

Figure 6 shows representative Raman spectra relative to the aforementioned TiO_2_ phases, focusing on the 180–800 cm^−1^ range, the most suitable shift interval for distinguishing the different TiO_2_ polymorphs^20^. The results of band component analysis are summarized in Table 2.

**Table 2:** Results of the Raman spectra band analysis concerning single grains and aggregates of TiO_2_. Values highlighted in bold correspond to the highest intensity in the mixed-phase spectra contain rutile and quartz.

*Single TiO*_*2*_ *grains*		
Band shift (cm^−1^)	Band assignments	Phase
206	*A*_1g_ Si-O symmetric bending vibrations	Quartz
**241**	**Combination line**	**Rutile**
404	*E*_g_ Si-O double stretching vibrations	Quartz
**441**	***E***_**g**_ **Ti-O double bending vibrations**	**Rutile**
466	*A*_1g_ Si-O symmetric stretching vibrations	Quartz
**609**	***A***_**1g**_ **Ti-O symmetric stretching vibrations**	**Rutile**
695	*E*_g_ Si-O double bending vibrations	Quartz
*TiO*_*2*_ *crystal aggregates*		
Band shift (cm^−1^)	Band assignments	Phase
197	*E*_g_ Ti-O double bending vibrations	Anatase
393	*B*_1g_ Ti-O antisymmetric bending vibrations	
516	*A*_1g_ + *B*_1g_ Ti-O symmetric and antisymmetric bending vibrations	
638	*E*_g_ Ti-O double stretching vibrations	

The Raman spectra of single TiO_2_ grains, as shown in Fig. 6a, displays a set of 4 peaks in the regions around 230, 441, 609 and 695 cm^−1^. The band analysis of the wide 230 cm^−1^ peak yields the combination of a 241 cm^−1^ band with a weaker at 206 cm^−1^, ascribed to the combination line of rutile and the *A*_1g_ Si-O symmetric bending vibrations of *α*-quartz, respectively, according to the literature^47–49^. The peak at 441 cm^−1^ corresponds to the most intense one in the analyzed wave number range, displaying a wide morphology and a shoulder to higher shift values. Band analysis of this peak reveals a combination of lower intensity bands at 404 and 466 cm^−1^ with the 441 cm^−1^ higher intensity band. The former can be assigned to *E*_g_ Si-O double stretching and *A*_1g_ Si-O symmetric stretching vibrations in quartz, respectively^49^, whereas the later corresponds to the *E*_g_ Ti-O double bending vibrations in rutile^47^. Finally, the bands at 609 and 695 cm^−1^ can be attributed to rutile’s *A*_1g_ Ti-O symmetric stretching vibrations^47^ and quartz’s *E*_g_ Si-O double stretching bending vibrations, respectively^49^. In Fig. 6b a representative Raman spectra of interstitial fine-grained aggregates of TiO_2_ is depicted. Here, a set of 4 peaks has been analyzed for band deconvolution. In order of increasing intensity, the bands at 638, 393, 516 and 197 cm^−1^ are in good agreement with reported shifts for O-Ti-O *E*_g_ double stretching, *B*_1g_ anti-symmetric bending, *A*_1g_ + *B*_1g_ symmetric and anti-symmetric bending vibrations, and *E*_g_ double bending vibrations, respectively, in anatase^50^.

**Figure 7:**
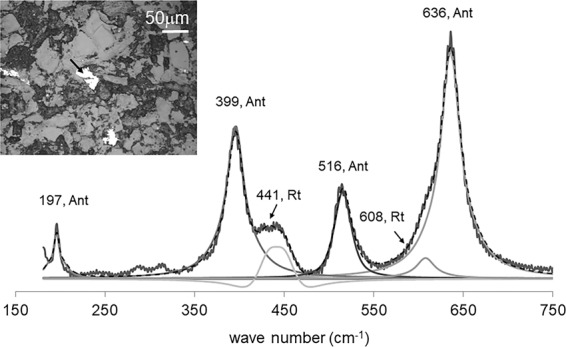
Representative Raman spectra of a single TiO_2_ grain displaying bands assignable to O-Ti-O bond vibrational modes of both rutile and anatase phases. The arrow displayed in the back-scattered electrons micrograph indicates the point of spectra acquisition. Rt=Rutile; Ant=Anatase.

There were few instances when the Raman spectra gathered for single TiO_2_ grains displayed bands assignable to both rutile and anatase’s O-Ti-O bond vibrational modes, as illustrated in Fig. 7. These results should be approached with caution, regarding the predominance of either phase when assessed with μRaman. In fact, the peak intensity recorded in Raman spectra is mostly dependent on the geometry of the experiment and the bond architecture of the analyzed substance. Therefore, a suitable orientation between incident radiation and bond polarization direction may result in higher intensity Raman band shifts, regardless of the bearing phase concentration in a mixture.

A striking feature in results of the band analysis is the slight decrease in vibrational frequencies with respect to the reported values for both rutile and anatase, except for the combination line at 241 cm^−1^ for rutile, and the *E*_g_ Ti-O double bending vibrations of anatase at 197 cm^−1^. It is worth mentioning that reference works^47–50^ display results of Raman analysis of synthetic, pure TiO_2_ single crystals, whereas this work focuses on naturally-occurring mineral phases which contain impurities in their crystalline structure. The relationship between non-stoichiometric compositional variations and band shift displacements is well documented in the literature^51–53^, providing tools for the interpretation of the present results, included in section 5.1.

Finally, very few single sub-rounded TiO_2_ grains, displaying reddish hews under reflected plane-polarized light, alongside the previously described sets of peaks typical of rutile band shifts, include bands at 300, 555 cm^−1^ attributed to goethite, and 224, 229, 408, 608 cm^−1^ ascribed to hematite band shifts^54–56^. The effects of heat-induced transformations of goethite into hematite and further broadening of bands, alongside indications of maghemite being present^56^ were not detected in our measurements. We interpret this fact as a result of the application of low laser power during Raman analysis.

### Chemical characterization of TiO_2_ phases

Table 3 displays the chemical contents in ppm of thirteen TiO_2_ isolated grains and five fine-grained aggregates relative to Si, Al, Fe, Cr, Ta, Zr and Nb, obtained with EMP. These results are further subdivided according to hosting formation, and the Nb/Ta ratios are shown alongside.

**Table 3:** Electron Microprobe analytical results in ppm for the studied TiO_2_ phases, where “b.d.” stands for “below detection”.

Formation	Analysis no.	Occurrence	Si	Al	Fe	Cr	Ta	Zr	Nb	Nb/Ta
Fara Fm	1	Single grain	4060	1540	2240	130	500	2340	480	1.0
	2	Single grain	920	b.d.	330	1470	b.d.	270	b.d.	-
	3	Single grain	460	b.d.	520	110	620	b.d.	1060	1.7
	4	Single grain	5550	3150	720	b.d.	570	720	640	1.1
	5	Single grain	1870	260	b.d.	300	530	1450	340	0.6
	6	Single grain	5160	1920	330	490	430	400	b.d.	-
	7	Single grain	b.d	b.d.	480	1220	880	650	1800	2.0
	8	Single grain	2320	b.d.	450	520	500	b.d.	2540	5.1
	9	Fine aggregate	11990	4020	650	840	580	b.d.	4290	7.4
	10	Fine aggregate	710	b.d.	970	110	1030	b.d.	7320	7.1
	11	Fine aggregate	11950	6020	1270	b.d.	930	570	7690	8.3
	12	Fine aggregate	15700	8950	1080	100	790	b.d.	10340	13.1
	13	Fine aggregate	2650	b.d.	800	b.d.	850	420	7580	8.9
Saiq Fm	14	Single grain	860	b.d.	410	b.d.	510	390	230	0.5
	15	Single grain	2210	290	550	b.d.	770	b.d.	460	0.6
	16	Single grain	430	b.d.	240	b.d.	870	b.d.	4030	4.6
	17	Single grain	280	b.d.	220	1260	610	720	1920	3.1
	18	Single grain	2340	740	640	150	210	1230	160	0.8

The low abundance of individuals, general small grain size (*d* ≤ 10μm), and frequent intergrown occurrence, posed analytical challenges to gather single-phase EMP data. Thus, in most cases, the acquired measurements include minor fractions of chemical constituents belonging to quartz micro-inclusions and/or surrounding quartz grains, with Si yielding 20 ≤ ppm ≤ 15700 concentrations, and maximum of 4.7 at.%. Considering the Raman profile revealed in Fig. 6a, the isolated grains of rutile clearly contain micro-inclusions of quartz, contributing to the measured Si and Al concentrations, alongside analyses from the surrounding grains. The representative Raman spectra displayed in Fig. 6b is free from peaks ascribed to Si-O bond vibrational modes, and only those of anatase occur. Therefore, Si and most of Al measured in fine aggregates of anatase conform to contamination exclusively arising from the surrounding media. These apparent contradictions are merely an artifact of the different analytical areal resolutions of the used μRaman (*d* ∼ 1μm) and EMP (5 < *d* < 10 μm) equipment, under the described analytical conditions.

Minute cracks and pores occasionally filled with Fe oxy-hydroxides, as indicated by the performed μRaman study, give rise to Fe concentrations ranging from 80 to 2240 ppm, whereas Al concentrations range from below detection levels to 8950 ppm.

These results clearly indicate that the analyzed TiO_2_ phases have been subjected to different degrees of dissolution, originating porosity in which secondary minerals of quartz, hematite and goethite may occur. Nonetheless, the results gathered for Nb, Ta and Zr can be exclusively ascribed to the targeted TiO_2_ phases, since such elements are excluded from the composition of quartz, whereas the measured concentrations of Fe (maximum 0.35 at.%) point towards negligible amounts of Fe oxy-hydroxides being present. Furthermore, the results shown in Table 3 do not reflect quantitative correlations between Fe or Al and Cr, Ta, Zr, and Nb concentrations, although the coupling of Al and Si in quartz is obviously patent. It is worth mentioning that rutile dominates Nb and Ta budgets in most samples, showing Nb/Ta ratios that should be identical to those displayed by the host rock’s^4,5,57^. In short, the contents of quartz and Fe oxy-hydroxides, alongside sample porosity, lead to a “dilution” of measured absolute concentrations of trace elements, while their relative ratios are retained.

The trace element compositions displayed in Table 3 reveal highly varying contents in Nb (below detection limit of ~102 to 10340 ppm) and Zr (below detection limit of ~160 to 2340 ppm), and less so in Cr (below detection limit of ~68 to 1470 ppm) and Ta (below detection limit of ~170 to 1030 ppm). Consequently, Nb/Ta ratios are also highly variable, yielding values in the 0.5 ≤ Nb/Ta ≤ 13.1 range, well below the chondritic value of 17.65^58^.

The occurrence of fine zircon lamella in the analyzed grains could be an undesirable source of Zr in our results, as shown by^59,60^. Nevertheless, the performed μRaman study did not reveal any evidence for the presence of zircon, especially the conspicuous band at ~358 cm^−1^ ^61^, always visible regardless of the geometry of the Raman experiment. This last observation is in agreement with previous optical inspections of the analyzed areas, which were also free from signs of chemical zoning patterns when checked under BSE mode. Finally, none of the analysis revealed abnormally high contents of Zr, even for Si concentrations> 300 ppm, following published data set exclusion criteria^11,62^ regarding the application of Zr-in-rutile single-phase thermometer functions.

## Discussion

### Cation substitution in TiO_2_

The results obtained from EMP analyses indicate that several trace elements with different valences substitute for titanium in the studied TiO_2_ phases. These replacements should occur in the lattice site of Ti^4+^, since the Raman data revealed band displacements towards lower wave numbers with respect to published data regarding pure rutile and anatase, a spectroscopic feature commonly found for solid solution systems^51–53^. Clearly, the assurance of charge neutrality is a major factor controlling cation incorporation in the structure of TiO_2_, as previously shown for rutile^1,2,63^. For example, the results depicted in Table 3 point towards the inclusion of trivalent Cr^3+^ and, possibly, Fe^3+^ and Al^3+^, together with pentavalent Nb^5+^ and Ta^5+^ in the structure of analyzed grains. Therefore, simple coupled-substitutions, such as Fe^3+^ + Nb^5+^ ↔ 2Ti^4+^, do not accurately reflect the overall ion replacement mechanisms in a complex multiple-end membered solid solution system. In reality, these replacements are also controlled by the ionic charge/radius ratios^64^, the inherent thermodynamic constraints of the solid solution system^5,65–67^ and the relative mobility of cations of interest in fluids. According to the findings reported in Vlassopoulos *et al*.^68^, a generalized equation for these substitutions seems more suitable:

1 $${X}^{3+}+{Y}^{5+}\leftrightarrow 2{{\rm{Ti}}}^{4+}$$

where, in the present case, *X*^3+^ corresponds to Fe^3+^, Cr^3+^, and Al^3+^, and *Y*^5+^ stands for Nb^5+^ and Ta^5+^. The data acquired in the present work does not allow an assessment of substitutions involving divalent cations. Nevertheless, the petrographic observations and Raman results provide strong evidence for oxidative reactive conditions, with Fe oxy-hydroxides ubiquitous in the samples, sometimes as micro-inclusions in TiO_2_, suggesting reaction (1) as the most likely coupled-substitution process. Likewise, the incorporation of interstitial H^+^ to compensate excess trivalent cations^3,68^ is out of the scope of the present study. Finally, Zr^4+^ (*r* = 0.72 Å) may directly replace for Ti^4+^ (*r* = 0.67 Å) in the crystalline lattice of the investigated phases, as demonstrated by Lippens *et al*.^32^.

Arbiol and co-workers^37^ discussed the charge balance constraints associated with the inclusion of Nb in the structures of both Nb-doped rutile and anatase. Their experimental study further proposes two charge balance mechanisms for Nb^5+^ uptake in TiO_2_, through one cation vacancy per four Nb introduced, or the reduction of one Ti^4+^ to Ti^3+^ compensating each Nb introduced. In spite of not being mutually exclusive, the latter process is typical of high temperatures, unrelated to the environmental conditions suggested by the presently studied mineral suites. Given the chemical similarities between Nb and Ta, it may be speculated that the substitution mechanisms proposed by Arbiol and co-authors should also apply to the latter element.

### TiO_2_ reactive pathways

Results of the performed Raman study indicate that the two TiO_2_ polymorphs, rutile and anatase, occur together in samples from WBA. Moreover, as Fig. 7 illustrates, they can be intergrown in close spatial relationship, whereas rutile single grains are frequently porous, with corroded borders, and anatase is always fine-grained, occupying interstitial spaces. These textural relationships suggest that anatase precipitation followed the solubilization of rutile substrate contents, in a coupled polymorphic dissolution-precipitation process. In fact, Smith *et al*.^38^ related the occurrence of anatase in terrestrial environments to low-temperature aqueous environments, associating its smaller grain size compared to rutile with the occurrence of structural hydration and/or adsorption of other surface species. This statement is in agreement with the observation of hydrothermal alteration products in our samples.

**Figure 8:**
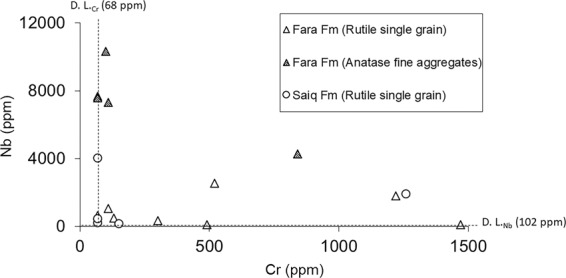
Nb vs. Cr contents in ppm of the analyzed TiO_2_ single grains and fine aggregates hosted by Fara and Saiq Formations.

**Figure 9:**
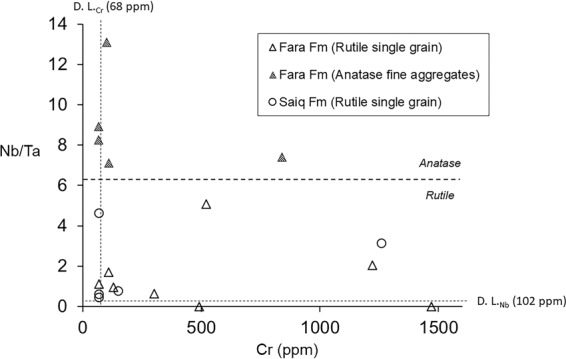
Na/Ta vs. Cr (ppm) of the analyzed TiO_2_ single grains and fine aggregates hosted by Fara and Saiq Formation.

Figure 8 displays Nb versus Cr concentrations in ppm of the analyzed TiO_2_ phases belonging to rock samples of the Fara and Saiq Formations. This type of chemical relationship has been used in past investigations as a discriminant tool for the visual assessment of rutile provenance in sedimentary clastic rocks, by effectively sorting data between metapelitic (i.e. felsic) and metamafic (i.e. mafic) sources^9–11^. Our results involve some concentrations below the analytical lower detection limit for either Cr (~68 ppm) or Nb (~102 ppm), but none of the measurements return Nb and Cr values simultaneously below the aforementioned thresholds. In spite of the underestimation of absolute concentrations described in section 4.3, Fig. 8 indicates that the anatase fine-aggregates contain strikingly higher concentrations of Nb than single rutile grains from both Fara and Saiq Formations. Cr concentrations vary widely, not displaying any correlation with Nb contents or the mineral mode of occurrence. These results provide further evidence pointing towards a coupled dissolution-precipitation process giving rise to the polymorphic TiO_2_ assemblages found in samples from WBA, accounting for the differential aqueous mobility of Nb and Cr, alongside their compatibility with rutile and anatase^20,37^. Thus, following the dissolution of rutile, Nb tends to incorporate preferentially in the newly-precipitated anatase, while solubilized Cr is more evenly scattered throughout the alteration products. This simple observation can be further explored by considering the relative enrichment in Nb of the analyzed TiO_2_ phases, as depicted in Fig. 9. Here, instead of Nb concentrations, the ratio Nb/Ta is used, providing a measure of Nb enrichment with respect to a similarly compatible pentavalent cation. Again, in spite of the high dispersion of data with respect to Cr, there is a clear sorting between the higher Nb/Ta values of anatase fine-grained aggregates and the lower of single rutile grains. These results suggest a direction of Nb enrichment towards anatase in the rutile-to-anatase dissolution-precipitation reactions for WBA samples. Furthermore, even though Nb/Ta ratios are consistently sub-chondritic (i.e. <17.65^58^), they vary extensively, further supporting mobilization of Nb from rutile substrates (0.6 <Nb/Ta <1.7) to anatase precipitates (7.1 <Nb/Ta <13.1).

In spite of absent mentions in the scientific literature to naturally occurring Nb-rich anatase, several experimental studies have tackled the effects of doping this TiO_2_ polymorph with Nb^31,33,37^. Notably, Arbiol *et al*.^37^ have shown that the inclusion of Nb in anatase hinders both its transition to rutile, and the coarsening of its grain size. Regarding the widening of the thermal stability field of anatase, these authors suggest that the uptake of Nb^5+^ cations should be compensated by a decrease in the number of defective oxygen vacancies, thus further stabilizing the structure of anatase. In more general terms, the effect of dopants like Y, Cr and Ta on the kinetics and coarsening related to the anatase-to-rutile transition, was approached by Banfield *et al*.^69^.

Finally, there remains to be addressed the absence of fine-grained anatase in rock samples collected at Saiq Plateau locations. As described in section 4.1, these samples contain much lower amounts of rutile, compared to their counterparts from WBA. Therefore, the necessary supersaturation with respect to anatase may not have been achieved to cause its precipitation from aqueous fluids. The profusion of quartz and carbonate veins in lithologies from SP, from meso to microscopic scales, also indicates higher fluid-rock ratios affecting these rocks. Furthermore, there are differences in the types, intensity and prevalence of hydrothermal mineral assemblages affecting rocks from WBA and SP. For instance, carbonation is much more extensive in rock types from SP, whereas sericitization is more prevalent in samples from WBA. Thus, it is reasonable to assume that the latter involved low pH conditions, whereas the occurrence of chlorite replacements rimmed by coarse-grained carbonates in samples from SP together with pervasive carbonation, suggest a later alkaline aqueous environment. Tanis al.^70^ studied the aqueous solubility of rutile at P-T conditions related to subduction environments, concluding that fluid composition was the major factor controlling the stability of this polymorph. For instance, this investigation demonstrated that high aqueous concentrations of Cl^-^ and F^-^ sharply increase rutile solubility under the approached P-T ranges. In summary, the absence of anatase in samples from SP can be explained both by unfavorable saturation conditions and differences in fluid chemistry.

### Crystallization temperatures *vs* polymorphism and Nb content

The results gathered in the present investigation, considering the observed mineral paragenetic suites and textural relationships, point towards hydrothermal environmental conditions underpinning the TiO_2_ polymorphic reactive pathways. However, the assessment of crystallization temperatures by the application of available thermometric techniques, involves important challenges. Most notably, the use of Zr-in-rutile single-phase thermometers^11–13^ is here made difficult by the analytical constraints mentioned in section 4.3 and the low temperature deviations observed by Cabral et al.^18^ in hydrothermal rutile. Furthermore, in the present case, coupled reaction products of rutile dissolution are anatase, for which the relationship between Zr content and crystallization temperatures has not yet been studied. However, as demonstrated by Shulaker et al.^19^, even at low temperatures and hydrothermal conditions, Zr concentrations in rutile are still temperature-dependent, and the crystal chemical similarities between Zr-doped anatase and rutile^32^ allow speculating that such must be the case also for the former polymorph, in spite of some expectable deviations. For instance, a higher tolerance of the anatase structure to cation replacement with respect to rutile has been shown to occur by Arbiol et al.^37^, leading to an expectable overestimation of crystallization temperatures by applying Zr-in-rutile thermometers to anatase phases, alongside the discrepancies observed by Cabral et al.^18^.

Petrographic observations performed in samples from WBA demonstrate that those are zircon and quartz-bearing lithologies, and microscopic textural evidence has been found for hydrothermal silicification affecting these rocks, thus indicating thermodynamic disequilibrium with respect to quartz. However, the lack of quartz veins crosscutting the mineralogy from micro to macroscopic scales, suggests low fluid-rock ratios, and the extent of such disequilibrium is therefore chemically and spatially limited. Nevertheless, the zircon grains show eye-catching signs of dissolution, pointing towards limited equilibrium with respect to both quartz and TiO_2_, especially anatase, which is a precipitation product.

In spite of all the listed challenges and limitations, calculating crystallization temperatures for anatase and rutile assemblages from WBA Fara Formation samples provides valuable semi-quantitative insights on the evolution of TiO_2_ crystallization environment in these lithologies. Thus, the TiO_2_ crystallization temperatures in samples from WBA were calculated using the approach of Watson *et al*. ^12^:

2 $${{\rm{T}}(}^{\circ }{\rm{C}})=\frac{4470}{7.36\,-\,\log ({{\rm{Zr}}}_{{\rm{ppm}}})}-273$$

This thermometer is based on experimental data acquired at constant ~10 kbar with an error of ±20 °C, providing the best choice to avoid speculation about pressure conditions when the origin of rutile is unknown^20^. Moreover, Chen and Li^71^ showed that pressure only exerts a major influence over Zr intake by rutile at ultrahigh pressure. In fact, the thermometric expressions reported in Tomkins et al.^13^ reveal that pressure is an almost negligible factor when compared to the ln(Zr) term at lower to intermediate pressure conditions.

Table 4 displays the crystallization temperatures obtained for TiO_2_ occurrences in zircon- and quartz-bearing rocks from Fara Formation, outcropping in WBA alongside their corresponding Nb/Zr ratios. The single rutile grains yielded temperatures below the ~594 °C detection limit to a maximum of 847 °C, and 0.6 <Nb/Ta <1.7 ratios. Taking into consideration the dilutive analytical effects over absolute concentrations described in Section 4.3 (Si content, signs of dissolution), these temperatures should be underestimated, and the most likely origin of rutile is therefore a magmatic environment. Moreover, unlike typical hydrothermal rutile, the grains investigated in this study lack any type of chemical zonation features (sectorial, oscillatory, etc.)^72^.

**Table 4:** Crystallization temperatures for TiO_2_ occurrences in Fara Formation zircon-bearing lithologies from WBA. *Nb <102 ppm detection limit; **Zr <160 ppm detection limit.

Occurrence	Analysis no.	T(°C)	Nb/Zr
Rutile single grain	1	847	0.2
	2	634	<0.4*
	3	<594	>6.6**
	4	720	1.1
	5	792	0.6
Anatase fine aggregate	9	<594	>26.8**
	10	<594	>45.8**
	11	698	13.5
	12	<594	>64.6**
	13	671	18.0

Contrary to rutile, some of the calculated temperatures of anatase crystallization are clearly overestimated (> 600 °C), especially considering the textural and mineralogical data pointing towards hydrothermal conditions of formation for this phase. In fact, even the application of the Zr-in-rutile thermometer to hydrothermal rutile returns consistently higher temperatures than O-isotope fractionation temperatures between quartz and rutile^19^. Cabral *et al*.^18^ compared Zr-in-rutile crystallization temperatures of hydrothermal rutile with total homogenization temperatures gathered from microthermometric fluid inclusion measurements, determining an ~200 °C overestimation affecting the former method. Even though the data acquired in the present investigation does not allow a quantification of the magnitude of such temperature overestimation, Table 4 reveals that assuming a similar 200 °C deviation would indicate crystallization temperatures in the 470–498 °C range for the two clearly overestimated anatase values, more consistent with hydrothermal conditions. An additional source for temperature overestimation is the higher tolerance of the anatase structure for cation replacement^37^, implying a different relationship between temperature and Zr concentration, than the one reflected by Zr-in-rutile thermometer. The determination of a Zr-in-anatase thermometer is out of the scope of the present work, as it requires specific purpose experimental studies.

Finally, the much higher Nb/Zr ratios encountered in anatase and listed in Table 4, further support the Nb-enrichment features discussed in section 5.2. It is worth nothing that anatase with crystallization temperatures <594 °C, yield much higher Nb/Zr ratios than those formed at higher temperatures. However, the single grain yielding temperatures <594 °C and Nb/Zr> 6.6 may correspond to rutile partially recrystallized to anatase, in agreement with Raman measurements displaying bands assignable to both polymorphs occurring in the same grains (Fig. 7).

## Conclusions

The results obtained in this work enabled deciphering the reactive pathways of TiO_2_ polymorphs hosted in volcaniclastic rocks from Jebel Akhdar, Oman. Most notably, the gathered evidence points towards a coupled polymorphic dissolution-precipitation process leading to the transformation of rutile to anatase under LT hydrothermal alteration conditions. Such reaction is also accompanied by Nb-depletion in the dissolved rutile, alongside consequent Nb-enrichment in anatase, further stabilizing the latter phase’s structure. Semi-quantitative thermometric considerations suggest that rutile occurring in the studied rock samples is of magmatic origin, while Nb-uptake by anatase is more effective at lower crystallization temperatures.

The observations performed in this research underline the relevance of addressing the possibility of TiO_2_ polymorphism in rutile geochemical studies, especially when hydrothermal alteration is involved. Furthermore, future research should focus on developing suitable thermometric tools adequate for the assessment of anatase crystallization temperatures.

## Data Availability

The research data used to construct all graphical representations and tables can be downloaded from the public access repository EarthChem.org, under 10.26022/IEDA/111495.
